# Tanzanian malignant lymphomas: WHO classification, presentation, ploidy, proliferation and HIV/EBV association

**DOI:** 10.1186/1471-2407-10-344

**Published:** 2010-07-01

**Authors:** Amos R Mwakigonja, Ephata E Kaaya, Thomas Heiden, German Wannhoff, Juan Castro, Fatemeh Pak, Anna Porwit, Peter Biberfeld

**Affiliations:** 1 Immunopathology Lab., Cancer Center Karolinska (CCK), Department of Oncology-Pathology, Karolinska University Hospital Solna/Karolinska Institute, Stockholm, Sweden; 2 Department of Pathology, Muhimbili University of Health and Allied Sciences (MUHAS), Dar es Salaam, Tanzania; 3 Pediatric Clinic specialized on Oncology and Hematology, Otto-Heubner-Center for Pediatrics, Charité Campus Virchow-Klinikum, Berlin, Germany; 4 Cancer Center Karolinska (CCK) Core Facility, Department of Oncology-Pathology, Karolinska University Hospital Solna/Karolinska Institute, Stockholm, Sweden; 5 Department of Immunology, Semnan Medical University, Semnan, Iran; 6 Department of Pathology, Radiumhemmet, Karolinska University Hospital Solna, Stockholm, Sweden

## Abstract

**Background:**

In Tanzania, the International Working Formulation [WF] rather than the WHO Classification is still being used in diagnosing malignant lymphomas (ML) and the biological characterization including the HIV/EBV association is sketchy, thus restraining comparison, prognostication and application of established therapeutic protocols.

**Methods:**

Archival, diagnostic ML biopsies (N = 336), available sera (N = 35) screened by ELISA for HIV antibodies and corresponding clinical/histological reports at Muhimbili National Hospital (MNH) in Tanzania between 1996 and 2006 were retrieved and evaluated. A fraction (N = 174) were analyzed by histopathology and immunohistochemistry (IHC). Selected biopsies were characterized by flow-cytometry (FC) for DNA ploidy (N = 60) and some by *in-situ *hybridization (ISH) for EBV-encoded RNA (EBER, N = 37).

**Results:**

A third (38.8%, 109/281) of the ML patients with available clinical information had extranodal disease presentation. A total of 158 out of 174 biopsies selected for immunophenotyping were confirmed to be ML which were mostly (84. 8%, 134/158) non-Hodgkin lymphoma (NHL). Most (83.6%, 112/134) of NHL were B-cell lymphomas (BCL) (CD20+), of which 50.9%, (57/112) were diffuse large B-cell (DLBCL). Out of the 158 confirmed MLs, 22 (13.9%) were T-cell [CD3+] lymphomas (TCL) and 24 (15.2%) were Hodgkin lymphomas (HL) [CD30+]. Furthermore, out of the 60 FC analyzed ML cases, 27 (M:F ratio 2:1) were DLBCL, a slight majority (55.6%, 15/27) with activated B-cell like (ABC) and 45% (12/27) with germinal center B-cell like (GCB) immunophenotype. Overall, 40% (24/60) ML were aneuploid mostly (63.0%, 17/27) the DLBCL and TCL (54.5%, 6/11). DNA index (DI) of FC-analyzed ML ranged from 1.103-2.407 (median = 1.51) and most (75.0%) aneuploid cases showed high (>40%) cell proliferation by Ki-67 reactivity. The majority (51.4%, 19/37) of EBER ISH analyzed lymphoma biopsies were positive. Of the serologically tested MLs, 40.0% (14/35) were HIV positive, mostly with high (≥40.0%) Ki-67 reactivity.

**Conclusions:**

According to the 2001 WHO Classification, most subtypes are represented in Tanzanian ML. Extranodal presentation was common among MNH lymphoma patients who also showed high aneuploidy, tumor proliferation (KI-67) and EBER positivity. DLBCL was frequent and phenotype heterogeneity appeared similar to observations in Western countries suggesting applicability of established intervention approaches. HIV was apparently associated with high ML cell proliferation but extended studies are needed to clarify this.

## Background

Malignant lymphomas (ML) represent a spectrum of lymphoid neoplasms with varying prognosis [1] including non-Hodgkin lymphoma (NHL), Burkitt lymphoma (BL) and Hodgkin lymphoma (HL). MLs occur worldwide with an increasing incidence both in industrialized countries and Africa [1,2]. Lymphomas represent today an important cause of morbidity and mortality in sub-Saharan Africa, including Tanzania partly due to the HIV and AIDS epidemic [3-5].

The classification of NHL has changed over time through the Rappaport Classification developed before lymphoid cells were divided into B-cells and T-cells [6], the International Working Formulation [WF] based on clinical aggressiveness [6], the Kiel Classification (based on histological grade) [7], the Lukes and Collins Classification which separated B-cell and T-cell lymphomas by immunologic techniques [8], the Revised European-American classification of Lymphoid neoplasms [REAL] [4] and most recently, the World Health Organization [WHO] classification [9,10]. The later two classifications recognize three major categories, B-cell neoplasms, T/NK-cell neoplasms and HL [9]. For HL the Rye Classification has been used for many years although, now slightly modified in the WHO/REAL Classification system [11,12]. Various countries have tested the applicability and adopted the WHO classification [11,13]. In Tanzania the WF is still used and attempts to apply the WHO classification have so far not been documented. Geographic and racial-ethnic differences in relative frequency of various ML have been reported [5,9]. Such geographic and racial-ethnic differences may influence the adaptation of prognostication as well as therapeutic protocols/algorithms shown to be effective elsewhere. An update and re-appraisal of Tanzanian ML diagnoses and characteristics possibly peculiar to this country is therefore needed.

It is now well documented that ML in HIV and AIDS patients also called AIDS-related lymphomas (ARL) have distinct clinical features including frequent extranodal presentation, which has not been evaluated in Tanzanian ML patients before our current study [4,14].

New insights into the pathogenesis of ML are continuously gained with the development in cytogenetics, molecular biology and immunological techniques [9]. Thus, similar to other cancers, ML may present with genomic instability including specific translocations and numerical chromosomal abnormalities (aneuploidy) [15-17]. Furthermore, the ML association with different viral infections is now well established including the Epstein-Barr virus (EBV) [4,17,18], the human immunodeficiency virus (HIV) [4,17] and recently, Kaposi's sarcoma associated herpes virus/human herpes virus type 8 (KSHV/HHV-8) in primary effusion lymphoma (PEL) and multicentric Castleman's disease (MCD) y. However, the prevalence of such viral associations is still sketchy in sub-Saharan Africa particularly Tanzania and is elucidated in our current study [3-5].

Likewise, the diffuse large B-cell lymphoma (DLBCL) group is now no longer one entity but rather a spectrum of NHL with heterogeneous histopathology, genotype, phenotype and prognosis including germinal center B-cell like (GCB) and activated B-cell-like (ABC) lymphomas [23,24]. Thus, the expression of proteins related to germinal centre B (GCB) cell or activated B-cells (ABC) and that of apoptosis-regulating proteins have been found to be associated with clinical outcome [25] and in particular Bcl-2 expression is strongly related to poor prognosis [25].

Ki-67 expression by proliferating tumor cells is a well established prognostic marker of malignancies including ML [26,27]. Such prognostic markers are poorly documented in Africa including Tanzania [14] impeding the development of comparable prognostication and adaptation of corresponding therapeutic protocols emphasized by our current studies.

Furthermore, data on clinical presentation and demographic (sex and age) characteristics for Tanzanian ML are scanty [14]. Our recent report on ML tumor proliferation included only mean values for major ML subtypes [14]. Those data are extended by our current long-term study

## Methods

### Study area

The study was conducted in the Histopathology Unit of the Department of Laboratory Services at the Muhimbili National Hospital (MNH) in Dar es Salaam, which is also the teaching hospital for the Muhimbili University of Health and Allied Sciences (MUHAS). MNH is the national referral health care facility in Tanzanian with a bed capacity of over 1000 and receives biopsies from most of the country except the lake zone which has its own manned pathology department.

#### Clinical presentation

This was retrieved from clinical records and was determined by physical examination, chest radiography and abdominal ultrasonography during the study period.

### Biopsies and HIV serology

Archival diagnostic biopsy material collected at MNH between 1996 and 2006 (10 years) was reviewed for histopathology and analyzed by immunohistochemistry (IHC), flow-cytometry (FC) and *in-situ *hybridization (ISH) at the Immunopathology Lab (Karolinska University Hospital Solna, Stockholm). The fixation and tissue processing protocols remained unchanged during the study period. Biopsies were selected for IHC or FC depending on the basis of representative tissue material per block. Clinical notes and histological reports for all ML biopsies submitted for diagnosis during the same period were also evaluated. The clinical evaluation protocol did not change during the study period. HIV-1 antibody serology (ELISA) was performed at the Microbiology/Immunology department at Muhimbili University of Health and Allied Sciences (MUHAS) as previously described [14,28].

### Histology

Primary histological diagnosis on hematoxylin and eosin (H & E) stained formalin-fixed paraffin embedded (FFPE) sections was done as previously described, at MNH according to the International Working Formulation [4,28].

#### Histopathological re-evaluation

(H & E and immunoperoxidase assay) of available tissue blocks was done at Karolinska University Hospital Solna independently and by three pathologists (ARM, PB and AP) according to the 2001 WHO classification of lymphoid neoplasms [9]. Inter-observer discrepancies were resolved by all three pathologists sitting together. Out of the H & E-evaluated cases biopsies selected based on the histological ML group, tumor:stroma ratio and absent/minimal necrosis, were further evaluated by immunohistology, cytomorphometry, flow-cytometry and in-situ hybridization.

### Immunohistochemistry (IHC)

Immunostaining was done (Immunopathology Lab) by the avidin-biotin complex immunoperoxidase technique as previously described [4,14,28]. Briefly, tissue sections were mounted on SuperFrost^® ^slides (Menzel GmbH & Co KG, Braunschweigh, Germany) deparaffinized, rehydrated and boiled for antigen retrieval at 750W by microwave (6 min) in citrate buffer pH 6. Endogenous peroxidase activity was quenched by incubating the sections in 30% hydrogen peroxide in distilled water (30 min) at room temperature (RT) then followed by washing in phosphate-buffered saline (PBS) and incubation with 1:20 normal serum from the species of the secondary antibody and washing (PBS). The sections were incubated overnight at 4°C with primary antibody for human antigens including pan-leucocyte CD45 (clone LCA), B-cell CD20 (clone L26), Reed-Sternberg (RS)/Hodgkin (HC) cells CD30 (clone Ber-H2), the Ki-67 (MIB-1) proliferation marker as well as the polyclonal rabbit CD3 T-cell marker. For DLBCL sub-typing, the immunophenotype of the tumors classified as germinal-center B-cell like (GCB) or activated B-cell like (ABC) lymphomas was determined using mouse anti-human CD10 (SS2/36), MUM1p (clone MUM1p), BCL-6 (clone PG-B6p) and BCL-2 (clone 124) all obtained from DakoCytomation, (Glostrup, Denmark) were used. The BCL-6/CD10/MuM1p markers were used for phenotypic and the BCL-2/CD10/MuM1p antibodies for prognostic sub-grouping. Thus DLBCLs could be categorized into activated B-cell like (ABC) if they were CD10-/BCL6+/MUM1p+, CD10-/BCL6- or CD10-/MUM1p+ and germinal center B-cell like (GCB) if they showed CD10+, CD10-/BCL6+/MUM1p- or CD10-/MUM1p- immunophenotypes. Furthermore, DLBCLs were categorized into prognostic group 1 (favourable prognosis) including all BCL-2- cases, BCL-2+/CD10+ and BCL-2+/CD10-/MUM1p- and group 2 (poor prognosis) which included cases with BCL-2+/CD10-/MUM1p+ reactivity [25]. For primary antibody detection, the sections were incubated (30 min, RT) with anti-species (secondary) antibody and the avidin-biotin complex respectively, and later developed (visualized) with 3,3'-diaminobenzidine (DAB) chromogen (Sigma-Aldrich, St. Louis MO, USA) as previously described [4,14,28]. After PBS washing, the slides were lightly counter-stained with H & E, blued in running tap water (30 min), dehydrated in ascending grades of ethanol, cleared in two runs of xylene and mounted with coverslips using Mountex (Histolab Products AB, Göteborg, Sweden).

Negative controls included sections from tissues not expressing the respective antigen as well as substitution of the primary antibody by buffer. Positive controls included tissue sections (lymph nodes and tonsils) with known expression of the antigen under investigation. These controls were included in each experiment.

### Microscopic evaluation

A fluorescence microscope (Olympus BX60, Tokyo, Japan) with a digital camera (Sony DKC-5000, Tokyo, Japan) and various filter cubes was used to document bright field and fluorescence microphotography. Cells were scored on color micrographs in eight adjacent fields (256 × 190 μm each) of characteristic lesions and the mean count of DAB-positive cells for each primary antibody (marker) was calculated. Strength of CD20 reactivity was subjectively scaled from 1+ to 4+ depending on staining intensity, thus intensity ≤2+ was regarded as weak and intensity>2+ was regarded as strong). Picture processing and printing was done using Adobe Photoshop 7.0 (Adobe Systems Incorporated, San Jose, USA) and Microsoft-Power Point, 2003 (Microsoft Corporation, Redmond, WA, USA).

### Tumor sample selection, enucleation and flow-cytometric analysis (FC)

FC on extracted lymphoma and tonsillar nuclei was performed as previously described [29]. Selected, non-necrotic tumour regions containing ≥70% neoplastic tissue in 90 μm thick sections were dissected, deparaffinized, rehydrated and digested for 1 hour at 40°C with 0.1% *w/v *Sigma protease XXIV (Sigma P8038) [Sigma-Aldrich, St. Louis, MO, USA] in Tris buffer [0.1 M Tris, 0.07 M NaCl (pH 7.2) [Merck, Darmstadt, Germany]. The obtained free nuclei in suspension were stained for 30' with 6-diamidino-2-phenylindole (DAPI) solution (10 μM DAPI in 800 mM disodiumhydrogenphosphate) [Sigma D9542, Sigma-Aldrich, St. Louis, MO, USA] and evaluated for DNA content by flow cytometry (≥2 × 10^4 ^nuclei per histogram). For the FC analysis, a PAS II (Particle Analysing System)-cytometer (Partec, Münster, Germany) and a LSRII Flow Cytometer (BD Biosciences, San Jose, CA) were used. The ModFit Program (Verity Software House; Topsham, ME, USA) was used for cell cycle analysis. Ploidy [DNA index (DI)] of diploid and aneuploid ML cell populations was compared to normal tonsil cells as previously described [30,31].

### Cytomorphometric evaluation

Extracted ML and control tonsillar nuclei were stained with DAPI and mounted with Vectashield [Vector Laboratories, Inc. Burlingame, CA, USA] mounting medium for fluorescence microscopy on SuperFrost^® ^slides. The mean size (S) and pleomorphism (P) of nuclei extracted from the tonsils were used as unity and were termed S_0 _and P_0_. Thus relative nuclear size (RNS) was the percentage size in excess of S_0 _evaluated as S_0 _+ size increase ≤25% = S1, S_0 _+ increase of 26 - 50% = S2, S_0 _+ increase of 51 - 75% = S3 and S_0 _+ increase >75% = S4. Evaluation of nuclear pleomorphism was P1 = mild, P2 = moderate and P3 = high. Mitotic figure counts (MFC) were evaluated on routine H & E sections. High power field (HPF) refers to ×400 microscopic magnification.

### *In-situ *hybridization (ISH)

Epstein-Barr Virus (EBV) infection detection was done by automated *ISH *as previously described [32] (Pathology Cell analysis Lab, Cancer Center Karolinska) using a fluorescein (FITC)-conjugated oligonucleotide probe to EBV-encoded (EBER) transcripts on FFPE tissue sections optimized for use with Bond Polymer Refine Detection (DS9800) and Anti-Fluorescein Antibody (AR0833) on the Bond-max system (Leica Biosystems Nussloch GmbH, Nussloch, Germany) according to the manufacturer's instructions.

### Statistical analysis

Data was analyzed using Statistical Package for the Social Sciences (SPSS) [SPSS Inc., Chicago Ill]. The Fisher exact test was used for smaller sample sizes. P-values of ≤ 0.05 were considered statistically significant.

### Ethical considerations and MNH guidelines

These studies were approved by the MUHAS Research Ethics Committee and the Ethical Committee, Karolinska University Hospital Solna (Dnr 01-096).

Upon obtaining individual informed consent and upholding safety, confidentiality and privacy, patients and material at MNH are also available for education and research since it was established as a University Teaching Hospital by an Act of Parliament. A strictly confidential and coded specimen processing and evaluation was conducted. HIV screening was performed by clinicians upon informed consent in the respective in-/out-patient units.

## Results

### ML frequency and general demography

During the period of study, the histopathology unit at MNH received approximately 50,000 biopsies including about 7,000 tumors out of which a total of 336 histologically diagnosed (H & E) lymphoma cases consecutively collected were evaluated. In 311 of 336 cases the information on patient sex (males 63.3%, n = 197/311 and females 36.7%, n = 114/311) was available. In 281 of 336 cases the age of patients was known, including 107 (38.1%) children (≤18 years), 131 (46.6%) adults (19-54 years) and 43 (15.3%) elderly (≥55 years) (Table 1). The overall age ranged from 4 to 91 years with a mean 31 and median of 30 years respectively.

**Table 1:** General demographic characteristics of confirmed malignant lymphomas (ML) at MNH between 1996 and 2006

Category	Number of ML cases	Percent
**Available sex data**	311/330	94.2
Female/Male	114/197	36.7/63.3
**Available age data (years)**	265/330	80.3
Juveniles (≤18y)	107	32.4
Adults (19-54y)	131	39.7
Elderly (≥55y)	43	13.0
Mean age	31.03	na*

*na = not applicable

### Clinical presentation

Data on clinical presentation (based on physical examination, radiography, abdominal ultrasonography and endoscopy) was available in 265 patients excluding cases later ruled out as ML after histological and immunohistological review. Majority 64.9%, (172/265) of these ML had nodal and 35.1% (109) extranodal disease at diagnosis. Extranodal presentation was reported in just over half (50.9%, 54/106) of childhood ML cases and in 20.9% (9/43) of the elderly, which difference was statistically highly significant (P = 0.00635, Chi-square Test) [Table 2]. Anatomical sites of extranodal presentation included visceral (12.5%), bone (10.0%) and soft tissues (6.4%) while cutaneous, oral cavity, nasal and ocular in descending order were rare. Furthermore, evaluation of ML anatomical distribution showed that majority (57.7% (153/265) had supra-diaphragmatic, 27.5% (73/265) sub-diaphragmatic and 26.8% (71/265) disseminated lymphoma. However, no primary effusion lymphoma (PEL) or primary central nervous system lymphoma (PCNSL), were found in this cohort [Table 3]. Data on clinical presentation was available in 115 cases morphologically reviewed at the Immunopathology Lab [Table 4]. Differences in distribution of anatomical sites of disease presentation by sex were not statistically significant (p-value 0.4695, Chi-square Test) [Table 2].

**Table 2:** Anatomical presentation of ML by age-group at MNH from 1996-2006

Age (Years)	Anatomical presentation [No. (%)]		Total [No. (%)]	P-value
			
	Nodal	Extra-nodal		
**Young (≤18)**	52 (33.1)	54 (50.0)	**106 (40.0)**	**0.00635**
**Adults (19-54)**	71 (45.2)	45 (41.7)	**116 (43.8)**	
**Elders (≥55)**	34 (21.7)	9 (8.3)	**43 (16.2)**	

**Total**	**157 (59.2)**	**108 (40.8)**	**265 (100.0)**	

**Female**	58 (36.9)	45 (41.7)	**103 (38.9)**	**0.4695**
**Male**	99 (63.1)	63 (58.3)	**162(61.1)**	

**Total**	**157 (100.0)**	**108 (100.0)**	**265 (100.0)**	

NB: 265 is the number of confirmed ML whose clinical data was available

**Table 3:** Anatomical site of disease presentation at diagnosis among TZ ML patients at MNH from 1996-2006

Anatomical site	Number of ML cases	Percent
Lymph node	157	59.2
Soft tissues	17	6.4
Oral cavity	8	3.0
Skin	9	3.4
Bone	28	10.6
Viscera	35	13.2
Ocular	4	1.5
Nasal	7	2.6

**Total**	**265**	**100.0**

NB: 265 is the number of confirmed ML whose clinical data was available

**Table 4:** Anatomical presentation of ML at diagnosis (MNH) by their reviewed histological subtypes (1996 -2006)

Reviewed histological diagnosis	Anatomical Site [No. (%)]		Reviewed cases with available clinical data	Clinical data unrecorded	Sub-totals	Total reviewed ML
	Nodal	Extranodal	[No. (%)]	[No. (%)]	[No. (%)]	[No. (%)]
***DLBCL***	35 (64.8)	7 (25.0)	42	15	57 (50.9)	
***BL***	2 (3.7)	15 (53.6)	17	6	23 (20.5)	
Other BCL*	17 (31.5)	6 (21.4)	23	9	32 (28.6)	
**Total BCL**	**54 (83.1)**	**28 (80.0)**	**82**	**30**	**112 (83.6)**	112 (83.6)
**TCL**	11 (16.9)	7 (20.0)	18	4		22 (13.7)
**TOTAL NHL**	**65 (65.0)**	**35 (35.0)**	**100**	**34**		**134 (84.8)**
**HL**	10 (66.7)	5 (33.3)	15	9		24 (15.2)
**TOTAL REVIEWED ML [No. (%)]**	**75 (65.2)**	**40 (34.8)**	**115 (72.8)**	**43 (27.2)**		**158 (100.0)**

*BCL = B-cell lymphomas

### Histology and immunohistochemistry (IHC)

**Figure 1:**
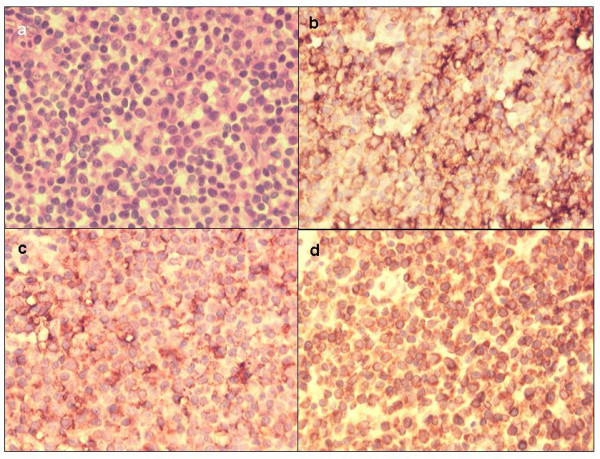
**(a, b, c, d)- Sections of an activated B-cell like (ABC) diffuse large B-cell lymphoma (DLBCL) case showing (a) H & E staining, and granular membranous and cytoplasmic immunoperoxidase (brown) reactivity in all tumor cells for (b) CD20 antigen, (c) BCL-6 antigen and (d) BCL-2 antigen (all ×400)**.

**Figure 2:**
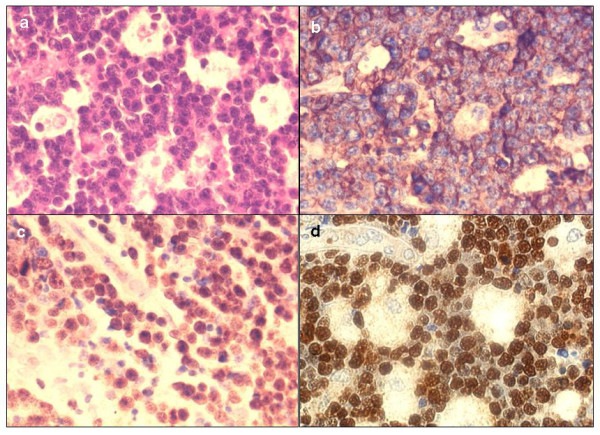
**(a, b, c, d)- H & E and immunoperoxidase staining (brown) of a BL case showing (a) H & E section with "starry sky pattern," (b) granular membranous and cytoplasmic reactivity in all tumor cells for CD20 antigen, (c) a high proportion of nuclear Ki-67 immunoreactivity (brown) and (d) EBER probe (dark brown) *in situ *hybridization (ISH) nuclear reactivity in all tumor cells, (all ×400)**.

**Figure 3:**
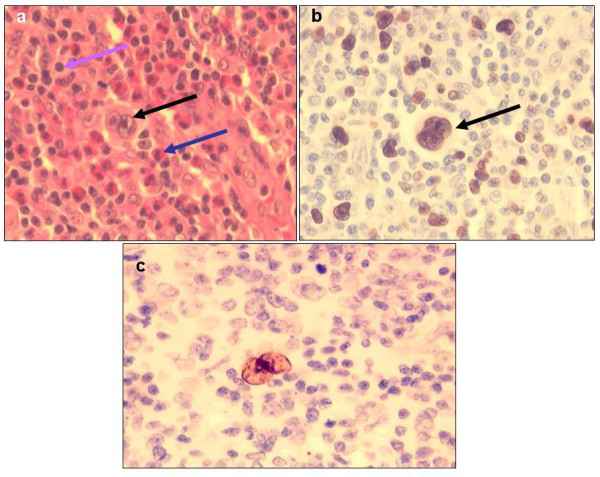
(a, b, c,)- HL histologic sections: (a) H & E staining of a classical HL mixed cellularity (MC) case showing Reed-Sternberg (RS) cells (black arrow), eosinophils (blue arrow) and lymphocytes (purple arrow), (b) nuclear Ki-67 immunoperoxidase reactivity (brown) in an RS cell, and (c) an EBER positive (brown) RS cell (all ×400).

A total of 174 biopsies out of the 336 cases above, were selected and stained by H & E and immunoperoxidase assay (IHC) based on availability of clinical notes, tissue blocks as well as quantity of material per block. After histopathological review and immunophenotyping using a panel LCA, CD20, CD3 and CD30 cell markers, a total of 158/174 were confirmed to be ML and 16 cases (9.2%) were excluded from further ML sub-classification, DNA ploidy and EBER studies but retained in the general study cohort to account for misdiagnosis. These represented mostly poorly differentiated metastatic carcinomas and sarcomas in lymph nodes as well as neuroendocrine tumors (carcinoids) but also a few cases of chronic inflammation including follicular hyperplasia and tuberculosis. Furthermore, the evaluation revealed 134 (84.8%) NHL, including 112 (83.6%) B-cell lymphomas (BCL) [CD20+, CD3-] and 22 (13.7%) mature T-cell lymphomas (TCL) [CD3+, CD20-] (Table 4). Most of the reviewed BCLs, (50.9%, 57/112) were DLBCL [Figure 1(a-d)], 20.5% (n = 23) were BL [Figure 2(a-d)] and 28.6% (n = 32) were other B-cell lymphomas [Table 4], which included small cell (SCL), lymphoplasmacytic (LP), follicular (FL) and marginal zone B-cell lymphomas (MZBCL) as well as plasmacytoma and oral plasmablastic lymphoma (OPBL). However, primary mediastinal large B-cell lymphoma was not found. TCL included peripheral T-cell lymphoma, extranodal NK/T-cell lymphoma (nasal type), angioimmunoblastic T-cell lymphoma as well as anaplastic large-cell lymphoma. Furthermore, 15.2% (n = 24) cases were HL (CD30+) [Table 4]. As expected, majority (53.6%, n = 15/28) B-cell lymphomas (BCL) with extranodal presentation were BL cases and most (80.0%, 28/35) NHL with extranodal presentation were TCL [Table 4]. Of the reviewed HL cases (Table 5), most showed a classical HL (CHL) histopathology) often of the mixed cellularity (MC) subtype [Figure 3(a)]. Furthermore, 6 (25.0%) cases were CHL nodular sclerosis (NS), 3 (12.5%) lymphocyte rich (LR) and 2 (8.3%) were lymphocyte depleted (LD). The non-classical nodular lymphocyte predominant Hodgkin lymphoma (NLPHL) subtype appeared rare (8.3%, 2/24) [Table 5] in this cohort.

**Table 5:** Histological subtypes of reviewed Hodgkin lymphoma (HL) cases from MNH between 1996 and 2006

Histological diagnosis	Number	Percent
**Classical HL (CHL)**		
Mixed cellularity (MC)	11	45.8
Nodular sclerosing (NS)	6	25.0
Lymphocyte rich (LR)	3	12.5
Lymphocyte depleted (LD)	2	8.3
**Non-classical HL**		
Nodular lymphocyte predominant (NLPHL)	2	8.3

**Total HL**	**24**	**100.0**

### DLBCL sub-typing

Of the 27 subtyped DLBCL (M:F ratio 1.7:1) cases, 3 (11.1%) were diagnosed in children and 24 (88.9%) in adults (Table 6). When sub-grouped for histological presence/absence of follicular structures most (74.1%) cases (n = 20) showed completely diffuse architecture and 25.9% (n = 7) had follicular remnants (Table 6). Using CD10, MUM1p, and BCL-6 markers together, we found that slightly more DLCBL in our series showed an ABC immunophenotype (55.6%, n = 15), by comparison to GCB immunophenotype 44.4% (12) although this was not statistically significant (P = 0.547, Chi^2 ^test) [Table 6]. As expected, all DLBCL with follicular remnants were GCB and most (75.0%) of those completely diffuse were ABC. The DLBCL immunophenotypes were associated with varying strength of CD20 reactivity and majority (57.1%, 12/21) of cases with strong reactivity were GCB (p = 0.02). Thus most (70.4%, 19/27) DLBCL examined were group 1 and 29.6% (8/27) were group 2 according to BCL-2/CD10/MuM1p sub-grouping (Table 6). In the present cohort, DLBCL subtype was not significantly correlated to age-group, HIV serostatus, tumor proliferation (Ki-67+) or DNA index/ploidy status which however, could depend on the small sample size.

**Table 6:** DLBCL (ABC/GCB) subtypes by clinical presentation, viral (HIV/EBV) status, IHC (Ki-67 and BCL-2) and DNA ploidy at MNH (1996-2006)

Subtypes [No. (%)]	Sex		Age-group		Clinical Presentation in relation to the diaphragm			HIV status		Ki-67 reactivity		EBER ISH reactivity			DNA Ploidy		BCL2/Mum1p subtype		Total [No. (%)]
	**M**	**F**	**≤18**	**>18**	**Supra-**	**Sub-**	**Gen**	**Pos**	**Neg**	**Low**	**High**	**Pos**	**Neg**	**nd**	**Diploid**	**Aneuploid**	**Grp. 1**	**Grp. 2**	**No.(%)**

**ABC**	9 (50.0)	6 (66.7)	0	15 (65.2)	9 (66.7)	4 (80.0)	4 (50.0)	2 (40.0)	1 (33.3)	11 (61.1)	4 (44.4)	5 (50.0)	8 (53.3)	2	6 (66.7)	9 (50.0)	7 (36.8)	8	**15 (55.6)**

**GCB**	9 (50.0)	3 (33.3)	3	8 (34.8)	3 (33.3)	1 (20.0)	4 (50.0)	3 (60.0)	2 (66.7)	7 (38.9)	5 (55.6)	5 (50.0)	7 (46.7)	0	3 (33.3)	9 (50.0)	12 (63.2)	0	**12 (44.4)**

**Total**	18 (66.7)	9 (33.3)	3 (11.1)	23 (88.9)	12 (44.4)	5 (18.5)	8 (29.6)	5 (62.5)	3 (37.5)	18 (66.7)	9 (33.3)	10 (37.0)	15 (55.5)	2 (7.4)	9 (33.3)	18 (66.7)	19 (70.0)	8 (30.0)	27* (100.0)

*This is the total number of DLBCLs analyzed by FACS for DNA Ploidy

nd = not determined

ABC = activated B-cell like DLBCL, GCB = germinal center B-cell like DLBCL

### Cell proliferation by Ki-67 immunohistochemistry and flow-cytometry (FC)

As expected, the highest (mean = 91.5%, range 75-98%) reactivity was found among BL cases [Figure 2(c)] followed by DLBCL (mean 42.4%, median 40.0%, range 10-90%, SD 23.5) and closely by TCL (mean 42.3%, median 50.0%, range 10-80%, SD 24.2) and lowest for HL [Figure 3(b)]. Furthermore, possible associations between Ki-67 index, sex, age or clinical presentation of ML cases as well as with that of different DLBCL subtypes, were not statistically significant. However, as expected, most (88.9%, 8/9) aneuploid ML cases showed high tumor proliferation rates (>40% Ki-67 reactivity) while only 11.1% of diploid ML had high tumor proliferation which difference appeared to be statistically significant (p-value 0.04, Fisher's Exact Test). FC S-phase Fraction (SPF) showed a mean for all ML of 19.6% (median = 16.5%) ranging from 3.8-39.7%. Comparison between Ki-67 index and SPF showed a significant correlation (R^2 ^= 0.55) between the mean SPF and Ki-67 reactivity.

### Epstein-Barr virus (EBV)

EBER *in-situ *hybridization (ISH) [Figures 2(d) &3(c)] was evaluated on 37 ML cases of which the majority (51.4%) were positive (19/37). Most (68.4%) of the EBER+ cases were found in the adult age-group (13/19) followed by juveniles (26.3%, 5/19) and rare in the group of elderly patients, which difference was statistically significant (p = 0.048). EBER reactivity was not correlated with either of the DLBCL immunohistological subtypes, p = 0.87 (not statistically significant, Chi-square test). Furthermore, EBER positivity correlation with sex, clinical presentation, tumor proliferation (Ki-67) and DNA index (DI) was also not statistically significant.

### Cytomorphometry results

**Figure 4:**
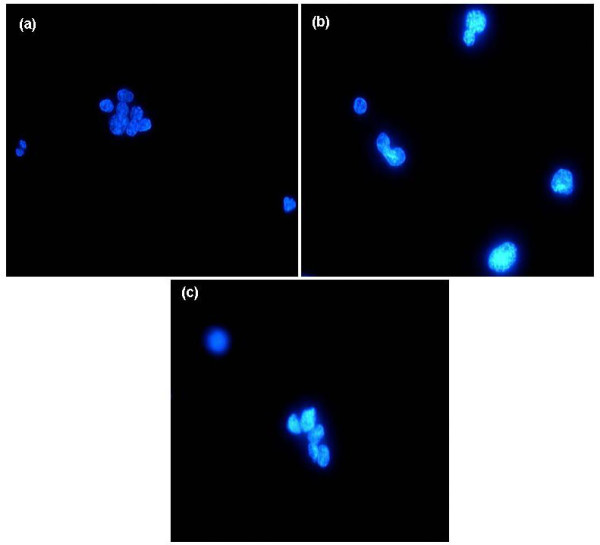
(a, b, c)- DAPI stained nuclei from a (a) normal paraffinized tonsil showing relative monomorphism and from DLBCLs showing pleomorphism (b & c) and mitoses (b).

DAPI-stained nuclei from 60 selected out of the 158 evaluated ML as well as from 3 tonsils were examined microscopically for changes in nuclear size, shape pleomorphism and cellularity (Figure 4) and as expected the highest nuclear size (S4) was most frequently seen in HL cases while a difference of nuclear pleomorphism (NP) and for DI between different ML groups did not appear to be statistically significant. Obviously, cellularity (number of nuclei/hpf) appeared significantly higher in BL followed by DLBCL, TCL and least in HL and appeared to correlate well with the median Ki-67 immunoreactivity.

### Flow cytometric (FC) results

**Figure 5:**
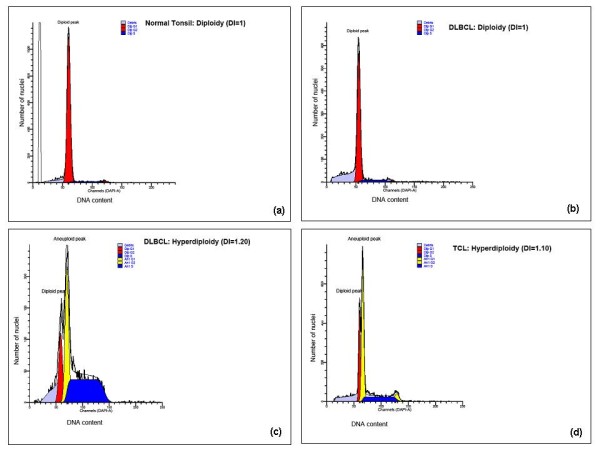
**(a, b, c, d)- DNA histograms of a diploid normal tonsil control (a), diploid (b) and hyperdiploid (c) DLBCLs as well as a hyperdiploid TCL (d)**.

**Figure 6:**
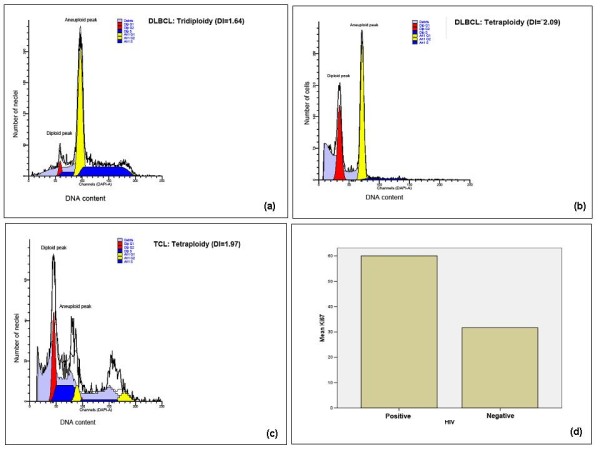
**(a, b, c, d)- DNA histograms of triploid (a) and tetraploid (b) Tanzanian DLBCLs, a tetraploid TCL (c), as well as a histogram of median Ki-67 reactivity (proliferation) and HIV serostatus in Tanzanian ML patients (d)**.

The nuclei extracted from the 60 selected cases evaluated by cytomorphometry above [including 27 DLBCL, 7 BL, 4 other B-cell lymphomas (BCL), 11 TCL and 11 HL] as well as those from three normal tonsils were analyzed for DNA index (DI) and tumor proliferation [S-Phase fraction (SPF)]. Microdissection and/or laser-capture microscopy (LCM) could not be done for HL cases in the current study due to logistical limitations. Thus, the HL ploidy results presented hereinafter are expected to serve as a proxy for HL cases at MNH. All the tonsillar controls were euploid or diploid (DI = 1) [Figure 5 (a)] but also some DLBCL [Figure 5(b)] and other BCL (including BL) as well as some TCL and most HL. Overall, 40% (24/60) of the selected ML cases showed DNA aneuploidy and the highest (63.0%, 17/27) frequency was seen in the DLBCL group followed by TCL (54.5%, 6/11) while aneuploidy was rare (14.3%, 1/7) in BL cases respectively, which differences were not statistically significant (p-value 0.062, Chi^2 ^test). Aneuploid DNA indices (DI) for tested ML biopsies ranged from 1.103 to 2.407 (mean = 1.65, median = 1.51, SD = 0.445). About 37.5% (n = 8) aneuploid cases were hyperdiploid (1 > DI < 1.3) mostly DLBCL (77.7%, 7/8) [Figure 5(c)] but also one TCL (12.5%) [Figure 5(d)] (p = 0.369, not statistically significant). Furthermore, only 12.5% (n = 3/24) of aneuploid ML cases showed triploidy (1.3 ≥ DI < 1.7) including two DLBCL [Figure 6(a)] and one BL whereas 50.0% were tetraploid/multiploid (DI ≥ 1.7) [12/24] including DLBCL (58.3%, n = 7) [Figure 6(b)] and TCL (41.7%, n = 5) [Figure 6(c)]. Thus tetraploidy appeared to be more (83.3%, 5/6) frequent among TCL cases compared to DLBCL (47.1%, 7/17) which difference was not statistically significant (p = 0.161, Fisher Exact Test).

### ML association with HIV infection (serology)

HIV screening was possible in only 22.2% (35/158) of the histologically reviewed ML cases of which 40.0% (14/35) were seropositive. Apparently, most (85.7%, 12/14) of the positive cases were seen in the younger adults (age 18-54). Furthermore, most (77.8%, 7/9) of the HIV seroreactive ML stained for Ki-67 showed high (≥40.0%) reactivity, which difference was statistically significant (p = 0.021, Fisher's Exact Test) [Figure 6(d)]. HIV association with sex, clinical presentation (disease extent and anatomic location), EBV infection (EBER+ ISH), and DNA index (ploidy) of ML cases appeared not statistically significant which could be due to small samples.

## Discussion

The ML demographics found in the studied, extended 10 year cohort are generally consistent with age and sex distribution of Tanzanian lymphomas discussed in our previous reports [4,14]. The finding that about one-third of the ML had extranodal presentation at diagnosis is of therapeutic importance and remarkably concordant with the 30-40% extranodal presentation observed among DLBCLs in Wurzburg, Germany [33] but not previously documented in Tanzania and in contrast to the frequency reported in Japan where extra-nodal presentation was higher (83.3%) in the young than (60.0%) in the older age-group [34]. Concordant with our findings in juvenile ML cases [34], nodal versus extranodal presentation is reportedly an important factor for determination of ML prognosis and pathogenicity related to genotypic and phenotypic differences in the two clinical groups which however is not well established [35]. Thus it seems that nodal and extranodal DLBCL, as well as DLBCL from different primary sites, are heterogeneous with regard to different biologic characteristics and prognostic implications [35]. Corresponding to other studies [36,37] we also found that TCL were more often associated with extranodal presentation particularly cutaneous and nasal compared to other ML subtypes [36,37].

Lymphoma classification remains a challenge particularly to resource-limited countries in Africa, where methods complementary to routine histology such as IHC, FACS, PCR and cytogenetics are mostly lacking. This is also the reason why the WHO classification has been difficult to implement in Tanzania. As realized from the present study combined immunophenotyping and H & E staining, clearly improved diagnostic specificity and should be implemented routinely. Thus the diagnosis of TCL by IHC in the current ML cohort to our knowledge is novel in Tanzania. Furthermore, the dominance of B-cell lymphomas, mostly DLBCL also reported in our previous study [14], and the proportions of BL, TCL and HL at MNH appear similar to other studies [9] suggesting the applicability of the WHO classification for Tanzanian ML.

The sub-phenotyping of African DLBCL by IHC using CD10, MUM1p and BCL-6 as well as BCL-2 cell markers in the present study has not our knowledge, been reported before in Tanzania and Africa. Thus, our findings indicate that previously established DLBCL sub-phenotypes also exist in Tanzania/Africa, which should allow the application of the same prognostication criteria and therapeutic protocols as those in developed countries. The apparently higher frequency of the ABC than GCB lymphomas, in the current MNH cohort appear to be concordant with several DLBCL studies from North America and Western Europe [23,25] although our sample size was small. However, this concordance may support the notion of pathogenic and biological similarity between Caucasian and African DLBCL heterogeneity. Our finding of architectural (histological) differences between diffuse DLBCL and those with follicular remnants suggests also the existence of DLBCL which may develop from nodal follicular lymphoma (FL) [38].

Furthermore, the application of the WHO classification of Tanzanian HL in the current study is novel including the reporting of classical (CHL) and the non-classical nodular lymphocyte predominant (NLPHL) cases. However, our HL findings of more frequent mixed cellularity (MC) than nodular sclerosing (NS) among cases with CHL, were not in agreement with reports from other countries were NS is reportedly more common [39,40] although other reports seem to concur with our current findings [11,41,42]. The reason for this discrepancy are not clear but the may possibly include the role of HIV infection in altering the epidemiology, pathogenesis and natural history of HL [41,42] considering that Tanzania is within the epicenter of the HIV and AIDS pandemic. However, these results could also depend on the sample size.

The 40% rate of aneuploidy found among the Tanzanian ML patients indicates a relatively high prevalence of genomic instability (chromosomal aberrations) and is in general in agreement with previous reports from Western countries [43,44] but higher than that observed among NHL in a Swedish report [16]. High DNA indices (triploidy, tetraploidy and multiploidy) were also found in our current study as well as a high mean DI comparable to other reports [16,45], which appears to correlate with biological aggressiveness and poor prognosis. The high DI (tetra-/multi-ploidy) and proportion of aneuploidy found most frequently among the current DLBCL cases is concordant with previous reports [45]. However, the lack of aneuploidy among our HL cases is in contrast to other reports [46] and could probably be due to the fact that microdissection/LCM for the neoplastic Reed-Sternberg cells (RSC) and/or Hodgkin cells (HC) was not done in our current study due to resource limitations as well as difficulty in suspending intact RSC known to be aneuploid. The strong correlation between aneuploidy and tumor proliferation (Ki-67) found in our study was expected as previously reported by others [47] and is indicative of a biological high tumor grade. There was no association between aneuploidy and HIV infection in our cohort but previous reports suggested higher proliferation and lower DI among HIV-associated lymphomas [44]. However, these reports are scanty, and further documentation is needed [48]. Furthermore, the finding that EBER positivity in our cohort, did not appear to be clearly associated with high tumor proliferation (Ki-67) could partly be due to the small sample size. Furthermore, the high frequency of EBER+ ML in our ISH-tested biopsies indicates a significant association of lymphomas with EBER positivity at MNH particularly in the adult age-group (68.4%) and is consistent with our previous report [4], in which we also showed co-expression (31.8%) of tumoral EBV-encoded RNA (EBER) and HHV-8 DNA (PCR) but not ML cell HHV-8 latency-associated nuclear antigen (LANA) association [4].

The finding that increased tumor proliferation in our current cohort appeared correlated to HIV infection, is concordant with previous reports [44] and supports the notion of viral-associated/driven tumor proliferation as a biological role in the oncogenesis of HIV-related malignancies similar to that reported for HHV-8 and Kaposi's sarcoma [28].

## Conclusions

The WHO Classification can apparently be applied for the diagnosis of lymphomas at MNH in Tanzania. Extranodal presentation of ML was frequent particularly for TCL. DLBCL subtype phenotype heterogeneity and frequency was similar to that observed in Western countries suggesting applicability of similar, diagnostic, prognostic and therapeutic approaches. Lymphomas at MNH appear to have frequent aneuploidy and EBER positivity as well as high DNA indices and tumor proliferation (Ki-67). HIV is apparently associated with increased ML cell proliferation but extended studies are needed to confirm this.

## Competing interests

The authors declare that they have no competing interests.

## Authors' contributions

ARM, PB, EK and AP designed research. ARM collected data. ARM, TH, JC, GW and FP performed research. ARM, PB, AP, TH, JC, GW, EK analyzed and interpreted data. ARM performed statistical analysis and wrote the manuscript. PB, AP, TH and EK corrected manuscript. PB contributed vital reagents and analytical tools. All authors read and approved the final manuscript.

## Pre-publication history

The pre-publication history for this paper can be accessed here:

http://www.biomedcentral.com/1471-2407/10/344/prepub
